# Development by Design: Mitigating Wind Development's Impacts on Wildlife in Kansas

**DOI:** 10.1371/journal.pone.0026698

**Published:** 2011-10-26

**Authors:** Brian Obermeyer, Robert Manes, Joseph Kiesecker, Joseph Fargione, Kei Sochi

**Affiliations:** 1 The Nature Conservancy, Cottonwood Falls, Kansas, United States of America; 2 The Nature Conservancy, Topeka, Kansas, United States of America; 3 The Nature Conservancy, Fort Collins, Colorado, United States of America; 4 The Nature Conservancy, Minneapolis, Minnesota, United States of America; 5 The Nature Conservancy, Boulder, Colorado, United States of America; Australian Wildlife Conservancy, Australia

## Abstract

Wind energy, if improperly sited, can impact wildlife through direct mortality and habitat loss and fragmentation, in contrast to its environmental benefits in the areas of greenhouse gas, air quality, and water quality. Fortunately, risks to wildlife from wind energy may be alleviated through proper siting and mitigation offsets. Here we identify areas in Kansas where wind development is incompatible with conservation, areas where wind development may proceed but with compensatory mitigation for impacts, and areas where development could proceed without the need for compensatory mitigation. We demonstrate that approximately 10.3 million ha in Kansas (48 percent of the state) has the potential to provide 478 GW of installed capacity while still meeting conservation goals. Of this total, approximately 2.7 million ha would require no compensatory mitigation and could produce up to 125 GW of installed capacity. This is 1,648 percent higher than the level of wind development needed in Kansas by 2030 if the United States is to get 20 percent of its electricity from wind. Projects that avoid and offset impacts consistent with this analysis could be awarded “Green Certification.” Certification may help to expand and sustain the wind industry by facilitating the completion of individual projects sited to avoid sensitive areas and protecting the industry's reputation as an ecologically friendly source of electricity.

## Introduction

Concerns over fossil fuel dependence and climate change have accelerated the development and deployment of renewable energy technologies in the United States. The U.S. Department of Energy (DOE) predicts that 20 percent of the nation's electricity could be generated from wind by 2030 [1]. Although wind energy is a relatively low-carbon source of energy, wind turbines have, per unit of energy produced, a larger terrestrial footprint than most other forms of electricity production [2]. Modern wind energy development requires approximately 20–28 ha per megawatt (MW) of installed capacity [1], and the ecological footprint of wind energy development can be even larger.

Depending on siting, wind energy may cause adverse impacts on wildlife, resulting in direct mortality to birds and bats, as well as habitat loss and fragmentation [3], [4], [5]. Although direct habitat losses from turbine footings and roads typically entail less than five percent of a wind energy project area, the habitat values of adjacent lands may be significantly diminished. Fragmentation is widely acknowledged to be detrimental to both the integrity of ecological systems and the long-term viability of associated wildlife [6], [7], and may act synergistically with climate change and other factors to magnify deleterious effects to species and ecosystems by limiting the ability of species to adapt or migrate [8], [9].

**Figure 1:**
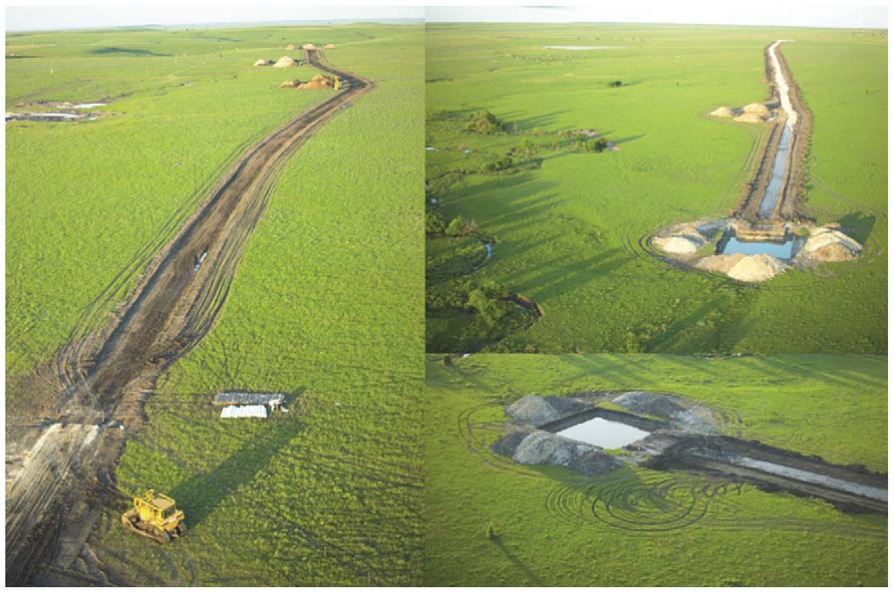
Road and turbine pads for a 150-MW facility in the Flint Hills of eastern Kansas. Over 30 km of new roads and 99 turbine pads were constructed in tallgrass prairie for this project.

Wind development projects may also result in fragmentation on a more local scale. At the 150-MW Elk River Wind Project near Beaumont, Kansas, nearly 30 km of new, improved roads were built across native tallgrass prairie to service the facility (Figure 1). Roads effectively fragment the habitat, restricting movement for many animals, possibly leading to population level impacts and genetic effects [10]. Edges of habitat caused by roads may also create an avenue for predators and invasive weeds and may affect fire behavior [11], [12]. While some bird species seem minimally affected by the presence of wind turbines [13], certain waterfowl, shorebird, and songbird species are known to avoid them. Grassland and shrubland-nesting birds are of particular concern, because these species are sensitive to human infrastructure and activity and may be evolutionarily disposed to avoid nesting and brood-rearing activities near vertical structures such as wind turbines [4]. Ongoing population declines for greater and lesser prairie-chickens and the intersection of their remaining distribution with some of the continent's prime wind generation regions compound the concern.

The DOE estimates that it will require about 5 million ha of land and nearly 18,000 km of new transmission lines in order for the U.S. to generate 20 percent of its electricity from wind [1]. Given the distribution of wind resources across the continental United States, certain states, such as Kansas, are likely to experience a disproportionate amount of development. According to DOE, however, wind energy production will require only about 3 percent of the land area with commercially viable wind resources in the continental U.S. This should allow ample opportunity to site wind energy development away from important and sensitive habitats.

Regulatory agencies often require that developers follow the mitigation hierarchy [14], which requires developers to avoid and minimize site impacts before utilizing offsets for negative impacts. We use a landscape-level approach to mitigation, referred to as “Development by Design” [15], [16], [17], which provides a quantitative approach to development that achieves no net loss for wildlife. Although the term “mitigation” has sometimes been used to refer only to the payments designed to offset or compensate for impacts, we restrict the use of “mitigation” to refer to the whole mitigation hierarchy sequence, which starts with avoidance of impacts. In the final step of the mitigation hierarchy, mitigation payments are required to offset or compensate for remaining impacts that cannot be avoided or minimized. We refer to this final step of the mitigation hierarchy as “compensatory mitigation” or “offsets.”

Our Development by Design approach to landscape-scale mitigation offers three distinct advantages over traditional project-by-project approaches: 1) it allows consideration of the cumulative impacts of current and projected development projects; 2) it provides a regional context to better determine which step of the mitigation hierarchy should be applied (i.e. avoidance versus offsets); and 3) it adds flexibility for choosing offsets to maximize conservation benefits by targeting the most threatened habitats or species. This method allows for mitigation funding to be pooled and allocated toward the highest conservation priorities, resulting in a higher conservation return on investment. This should lead to reduced development costs and improved conservation outcomes compared to project-by-project approaches to offset impacts.

Here we apply Development by Design to wind energy development in Kansas. This framework identifies areas where impacts to important habitats cannot be offset and, therefore, should be avoided. The framework also provides a method to identify areas where development may proceed without significant ecological concerns, as well as areas where ecological impacts will be significant, but can be offset. Importantly, this approach provides a mechanism to quantify expenditures necessary for offsets where they are appropriate. Finally, we discuss possible incentives to encourage the use of this framework.

## Methods

We next describe the scientific basis for the following recommendations: 1) which areas to avoid in Kansas, 2) how to quantify impacts that need to be offset, and 3) how to offset impacts. These recommendations form the basis for the GIS analyses and mitigation cost calculations presented in the results.

### Identifying Areas Where Wind Energy Development Should be Avoided

**Figure 2:**
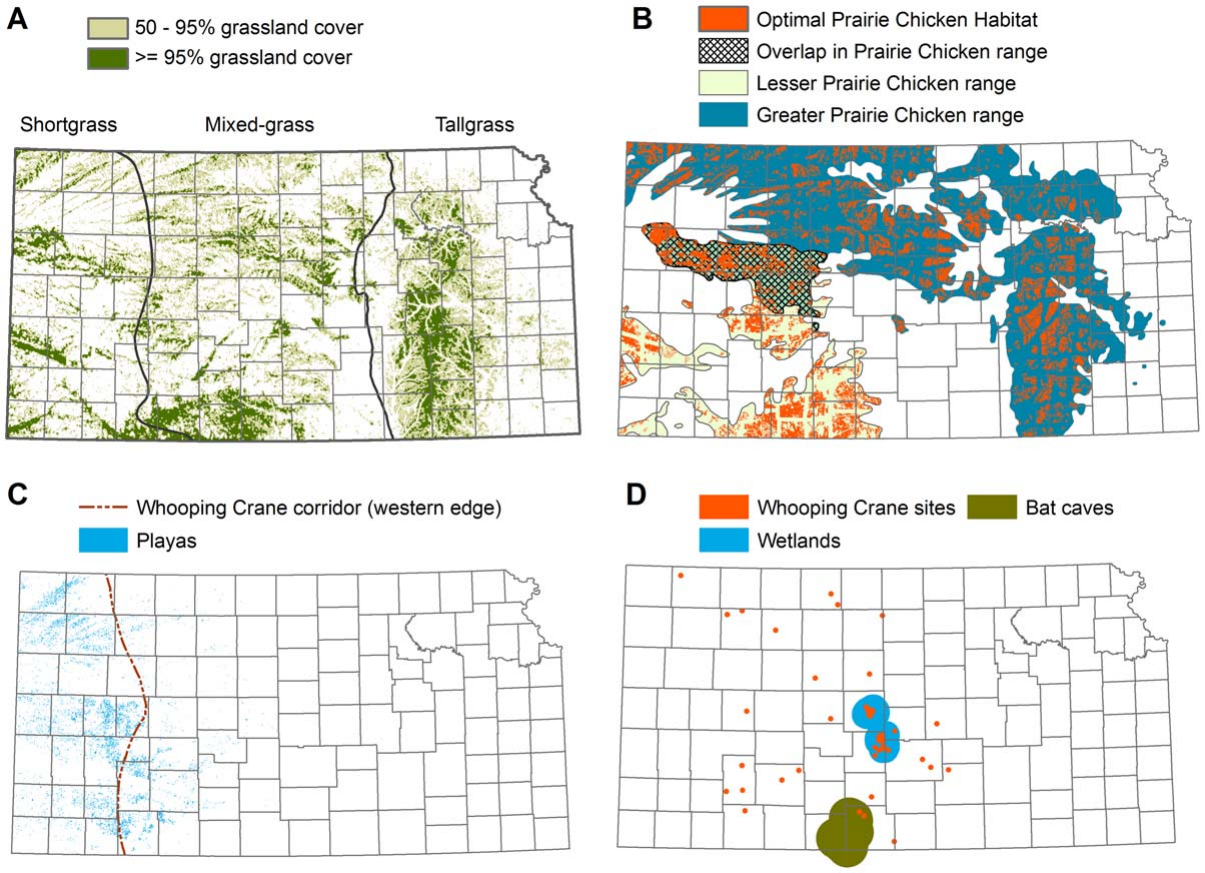
Key habitats in Kansas. A) Intact grasslands. Light green for grasslands 50–95% intact, darker green for grasslands >95% intact. Blue lines show boundaries for shortgrass, mixed grass, tallgrass ecoregions. B) Prairie-chicken range and optimal habitat for greater and lesser prairie-chickens. C) Playas and the western edge of the whooping crane migration corridor. D) Repeated whooping crane stopover sites, Cheyenne Bottoms and Quivira National Wildlife Refuge with a 16 km buffer, and Red/Gypsum Hills bat roosts with a 24 km buffer.

Kansas contains many unique habitats and associated wildlife populations. Our analysis follows best practices for conservation planning [18] by considering multiple conservation targets designed to preserve both whole landscapes and particularly sensitive areas (Figure 2; Table 1). Specifically, we include key habitats (intact grasslands and playas), umbrella species (greater and lesser prairie-chickens), imperiled species (whooping crane), and areas of wildlife congregation for taxa that may be vulnerable to wind energy impacts (bat roosts and playas). We make use of over a dozen pre-existing spatial datasets of habitat, land cover, land use, wind speed, protected areas, roads, transmission lines, wind turbines, soils, and species occurrence. Complete descriptions of methods and sources of error in these datasets is beyond the scope of this paper, but can be found in the relevant citations. However, each dataset is of sufficient quality for use in our landscape-scale assessment.

**Table 1:** Avoidance areas for wind turbines in Kansas.

Wind energy projects impacts cannot be mitigated if any turbine is located within:
1) 3.2 km buffer around wetlands with repeated whooping crane sightings (sites documented with repeated use: 3 days or more and/or multiple year sightings)
2) 800 meters of a “very high” quality playa lake within the whooping crane migration corridor; or 400 meters of a “very high” quality playa lake outside of the whooping crane migration corridor
3) 16 km buffer around Cheyenne Bottoms Wildlife Area and Quivira National Wildlife Refuge wetlands of international importance
4) 24 km buffer around a cluster of terrestrial caves in south central Kansas known to be important bat habitat
5) ≥95% native prairie and CRP (800 m radius moving window), with a 1.6 km buffer around the subset of these grasslands that are also optimal prairie-chicken habitat.
6) areas designated by the U.S. Fish and Wildlife Service (USFWS) or Kansas Department of Wildlife and Parks (KDWP) as having the presence or habitats of threatened or endangered species

Tallgrass prairie is the continent's most diminished ecosystem in terms of area lost, with only 4 percent of the original tallgrass prairie area remaining [19], [20], [21]. Although less impacted than tallgrass prairies, short and mixed-grass prairies have also experienced significant reductions throughout the Great Plains. Estimated state and provincial declines of native mixed-grass prairie range from 30 to 99 percent, and 20 to 85 percent for shortgrass prairie [19]. Consequently, large, intact native prairies are unique habitat types that cannot be replaced and are critical to sustaining population of several species, such as greater and lesser prairie-chicken. We recommend that areas with remaining grassland cover of greater than 95 percent intactness, as defined below, be avoided for wind energy development. Further, for the subset of these grasslands that are also considered optimal prairie-chicken habitat, we recommend that no turbines be placed within a 1.6 km surrounding buffer. This is based on 1) evidence that prairie-chicken avoid areas affected by habitat fragmentation, human activity, and the presence of vertical structures [22], [23], [24], [25], 2) data showing reduced nest success and fecundity of prairie chickens in proximity (<2.2 km) of a wind facility [26], 3) other species of grouse's avoidance of wind turbines [27] and oil and gas development [28], [29], and 4) expert opinion [4], [26], [30].

To effectively delineate intact grasslands, we first identified grasslands using data layers for CRP lands and warm season grasses from the 2005 Kansas Land Cover Patterns dataset [31]. We then applied a moving 800 m radius window to identify areas with remaining grassland cover exceeding 95 percent as “intact grasslands” (Figure 2A). We identified optimal prairie-chicken habitat [32] by first delineating potential habitat. Native prairie and CRP grasslands [31] within the known prairie-chicken range with greater than 50 percent grassland cover were considered potential habitat. Potential habitat was then smoothed using a 90 m×90 m moving window. The following areas [32] were excluded from potential habitat due to existing impacts: 1) primary and secondary roads with a 2,377 m buffer; 2) wind turbines and urban areas with a 1,600 m buffer; 3) oil and gas wells with a 564 m buffer; 4) electric transmission lines greater than or equal to 345 kv with a 500 m buffer; and 5) woodlands and a 161 m buffer. We then removed patches smaller than 518 ha and the remaining land was considered optimal prairie-chicken habitat (Figure 2B). The subset of this optimal prairie chicken habitat that occurs on >95 percent intact grasslands should be avoided by wind development.

In addition to the state's intact grasslands, there are several important areas of wildlife concentration in Kansas. Cheyenne Bottoms, the largest marsh complex in the interior United States, and Quivira National Wildlife Refuge together comprise one of the most important shorebird migration stopover sites in the Western Hemisphere [33], [34], [35]. Spring surveys indicate that up to 45 percent of the North American shorebird population may utilize these wetlands during northward migration in some years [34], [35]. Because the legal boundaries for these sites do not include adjacent areas of ecological importance, we recommend a buffer of 16 km around Cheyenne Bottoms and Quivira National Wildlife Refuge (Figure 2D). This buffer also addresses the fact that these two large wetlands and closely associated uplands are frequently used by whooping cranes and other species of concern. Whooping cranes, a federally listed species with approximately 380 individuals remaining [36], [37], depend on the Cheyenne Bottoms-Quivira wetland complex for survival during migration. Whooping cranes may be susceptible to collisions with turbines when landing, taking off, and travelling to foraging sites [37].

The U.S. Fish & Wildlife Service (FWS) has empirically defined a whooping crane migration corridor as the boundary within which 95 percent of all whooping crane stopover sightings have occurred. Areas with repeated whooping crane sightings represent the best empirically-based approach to predicting future use of sites by the species. Whooping cranes may be at risk of turbine collisions when ascending or descending from migration, or when making low flights from roost sites to foraging areas, which often extend for up to 3.2 km [38]. To address this concern, we recommend that areas within 3.2 km of repeated whooping crane stopover sites be avoided by wind energy development (Figure 2D).

In central and western Kansas, migratory birds rely on seasonal, shallow, clay-lined lakes, referred to as playas [39]. Playas differ in size, connectivity, and their surrounding land cover (e.g., grassland versus cropland). A recent assessment of playas in Kansas suggests that relatively few of the 22,000 playas in Kansas likely function at a very high level and that these playas should be the top priority for conservation (M. McLachlan, PLJV, personal communication). We recommend that playas of “very high quality” within the whooping crane migration corridor be avoided for wind energy development within 800 m, because whooping cranes are likely to avoid playas within 800 m of a turbine [38]. In general, playas provide important habitat for a wide range of birds in addition to whooping cranes (including migratory birds covered under the Migratory Bird Act). Therefore, we recommend that playas of very high quality outside of the whooping crane migration corridor be avoided within 400 m (Figure 2C) based on evidence that: 1) Anseriformes and Charadriiformes experience declines in abundance in proximity to wind facilities [40]; 2) European golden plovers and northern lapwings were displaced by as much as 600 m at a wind facility in Denmark [41]; and 3) Several European studies found up to a 95 percent reduction in birds up to 250–500 m away from wind turbines [13].

Bats, especially migratory tree-roosting species, have exhibited high mortality rates associated with wind turbines [42], perhaps because bats are attracted to wind turbines [43], [44] and bats can be killed by the pressure drop associated with wind turbines even without direct strikes from turbine blades [45]. In general, little information is available regarding the geographic distribution of bat roosting, foraging, and migration areas. However, a cluster of caves in the Red/Gypsum Hills are known to provide important habitats for bats [46]. Bats have been shown to forage out to 24 km from roosting sites [47], [48]. Pending additional research, we therefore recommend an avoidance buffer of 24 km surrounding known bat concentration sites in south-central Kansas (Figure 2D).

Finally, any area identified by the FWS or the Kansas Department of Wildlife and Parks as containing threatened and endangered species habitat or occurrences should be avoided for wind energy development. Each project area should be assessed in consultation with these agencies.

### Quantifying Impacts that Need to be Offset

Among the sensitive habitats described above, development and corresponding compensatory mitigation would be allowed only for impacts to grasslands and prairie-chicken habitat that is 50–95 percent intact and for playas of less than very high quality (Table 2). Other sensitive habitats described previously (Cheyenne Bottoms/Quivira National Wildlife Refuge, Red/Gypsum Hills bat caves, and repeat whooping crane stopover sites) should be avoided entirely. Therefore, offsets need not be calculated for them.

**Table 2:** Wind turbine mitigation areas and costs in Kansas.

Habitat type	Turbine ecological footprint buffer	Definition	Mitigation action per ha impacted	Cost per ha of impacted habitat
Grassland	1,600 m	50–95% intact grassland	Conservation easement on 1.67 ha of >95% intact grassland	Short grass: $825
				Mixed grass: $862
				Tallgrass: $1,432
Playa	400 m (800 m w/in whooping crane corridor)	high quality	Permanently protect of 1 ha of high or very high quality playa and 3 ha of surrounding watershed and restoration to grass	$12,872
Playa	400 m (800 m w/in whooping crane corridor)	medium quality	Permanently protect of 2/3 ha of high or very high quality playa and 2 ha of surrounding watershed and restoration to grass	$8,496
Playa	400 m (800 m w/in whooping crane corridor)	low quality playas that are part of a complex	Permanently protect of 1/3 ha of high or very high quality playa and 1 ha of surrounding watershed and restoration to grass	$4,248
Prairie-chickens	1,600 m	Short grass optimal prairie-chicken habitat	brush management and a 7-yr return interval prescribed fire endowment on 1 ha of optimal habitat	$246
Prairie-chickens	1,600 m	mixed grass optimal prairie-chicken habitat	brush management and a 5-yr return interval prescribed fire endowment on 1 ha of optimal habitat	$261
Prairie-chickens	1,600 m	Tallgrass optimal prairie-chicken habitat	brush management and a 3-yr return interval prescribed fire endowment on 1 ha of optimal habitat	$296

We calculate the area impacted for each habitat type by estimating the ecological footprint of each turbine. The ecological footprint differs among species and habitat types, due to differences in species' turbine avoidance behavior and direct mortality vulnerability. As discussed above, we use three distances from turbines to calculate ecological footprints. For impacts to intact grasslands and greater or lesser prairie-chicken habitat, the ecological footprint encompasses a 1.6 km radius from wind turbines [4], [22], [23], [24], [25], [26], [27], [28], [29], [30]. For impacts to playas outside the whooping crane migration corridor, a 400 m radius from wind turbines represents the ecological footprint [13], [40], [41]. For impacts to playas within the whooping crane migration corridor, the ecological footprint includes an 800 m radius from turbines [38].

### Methods for Quantifying the Amount of Offset Needed

Under existing policies, habitat impacts are commonly offset according to “replacement ratios” that specify how many habitat units must be replaced or protected for each unit impacted. However, replacement ratios are generally too inflexible to address the ecological context for impacts and offsets, and common alternatives are too subjective [49]. The accounting method we propose seeks a more repeatable and transparent approach.

An offset's contribution to no-net-loss goals depends on: 1) additionality (defined as an offset's new contribution to conservation, in addition to existing values); 2) the probability of success (defined as the likelihood that offset actions will deliver expected conservation benefits); and 3) time lag to conservation maturity (evaluated as the length of time required for offset actions to replace lost habitat values; e.g. time to maturity for ecological restoration). Note that our framework can be applied to both habitat protection and restoration efforts. When offsets restore degraded ecosystems, they provide new contributions to conservation over time as the offset reaches maturity. Habitat preservation also delivers added conservation value when, taking into account real-world conditions and threats, such preservation reduces an expected rate of loss. For example, protecting a 1,000-ha grassland that was experiencing conversion to cropland at an average rate of 1 percent per year would deliver a new contribution to conservation of 10 hectares per year (1 percent of 1,000 ha). Such “background” rates of loss can be estimated using a range of threat assessment approaches, e.g. [50], [51], [52].

#### Intact Grasslands

In order to conserve intact grasslands, offset projects should provide benefits equivalent to the area of grassland impacted by the ecological footprint of the project, which extends 1.6 km from each turbine. Compensatory mitigation should target preservation of large, intact grasslands that have 95 percent or greater grass cover (Figure 2A). Offsets containing similar ecological values, i.e. “in-kind offsets,” should be given preference. For example, impacts to short-grass habitats should be offset in a short-grass ecosystem (Figure 2A).

The scarcity of large intact prairie means that preserving the remaining occurrences, which are still at risk of conversion, is generally the top priority for conservation of grassland species [53]. Generally, restoration of native prairie is more expensive and less likely to provide diverse native habitat than protecting existing at-risk prairie with conservation easements. Conservation easements are the primary tool for preventing conversion of intact prairie by restricting future development rights in perpetuity. Common prohibitions in conservation easements include residential and commercial development, energy development and extraction, surface mining, and soil disturbance such as plowing. The per-ha value of a conservation easement is established by standard appraisal.

Targeting conservation easements towards existing prairie that is at risk of conversion can increase the amount of habitat remaining over time, compared to a scenario in which habitat losses continues unabated. Quantifying the “additional” benefit of prairie protection requires calculating the background rate of loss that is expected to occur in the absence of protection. We identify the rates of conversion, and the areas at risk for conversion, for three types of development in Kansas: wind energy development, exurban development, and cultivation.

#### Wind Development

**Figure 3:**
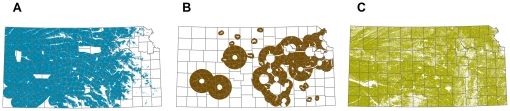
Ongoing development in Kansas. A) Areas of potential wind development, B) Areas of potential exurban development, C) Areas of potential cropland conversion.

We examined 32 proposed wind energy projects in Kansas [54] to characterize areas with significant wind development potential. Over 90 percent of proposed new generating capacity is located on lands with wind power class (WPC) of 3 or greater (measured by the NREL 50 meter wind power class data [55]), with a MW-weighted average WPC of 4.1 for all proposed new generation. For these projects, the farthest distance to transmission lines of 115 kV or greater was 25.5 km. We therefore considered areas with a WPC 3 and higher that are located within 25.5 km of current and proposed transmission lines of 115 kv or greater to have significant potential for wind energy development. We then excluded areas protected from wind development [56] and urban areas in which wind development is not feasible [57], [58] (Figure 3A). In the future, new transmission lines could increase this area, indicating the need to update estimates of background rates of loss as new information becomes available.

The DOE's “20% wind energy by 2030” report offers a scenario in which the U.S. could generate 20 percent of its electricity from wind power by 2030 [1]. Under this scenario, Kansas would have 7.16 GW of nameplate capacity (peak electrical output of all turbines running at capacity). Kansas currently has 1.03 GW of nameplate wind energy capacity, suggesting that between 2010 and 2030, Kansas could acquire approximately 6.13 GW of new wind energy capacity. This is the equivalent of developing 306.5 MW of capacity per year for 20 years. New wind energy development in Kansas can, therefore, be expected to require about 7,000 ha per year. Given that 14.5 million ha of Kansas has significant potential for wind energy development, we calculate that these areas have a probability of wind development of 0.05 percent per year.

#### Exurban Development

We used the Kansas Natural Resources Conservation Service (NRCS) map of areas susceptible to urban expansion [59] and the National Land Cover Database (NLCD) 1992–2001 Land Cover Change Retrofit product [57], [60] to estimate the rate of residential development. The NRCS estimates that the following areas in Kansas are at risk of exurban development: 1) within 48 km of metropolitan areas greater than 19,000 people; 2) within 24 km of other metropolitan areas; and 3) within 8 km of federal reservoirs. After excluding areas already protected from development and those already converted to urban uses, we estimate that there are 6.7 million ha at risk of conversion to exurban development in Kansas (Figure 3B). The NLCD change product allows us to quantify the land area that changed from other land cover categories to the “developed” category between 1992 and 2001. Within the areas we identified to be at risk of exurban development, the NLCD change product estimates that 12,500 ha were urbanized between 1992 and 2001. This is a rate of 1,400 ha per year, or 0.02 percent of the susceptible area per year.

#### Cropland Conversion

We used USDA's land capability class [61] to estimate the areas at risk of conversion to cropland. USDA categorizes land into eight capability classes, with classes 1–4 described as suitable for cropland, and classes 5–8 described as unsuitable for cropland. We identified class 1–4 lands in the state, excluding areas that have already been converted to cropland (based on 2006–2008 NASS data [62]), lands that are protected from conversion [56], and lands that have been developed for residential and commercial uses (based on NLCD “developed” layers [57], [58]). Based on this analysis, there are 5.2 million ha of potential cropland in Kansas that have not yet been cropped (Figure 3C). We used the NASS 2009 cropland data [62] to calculate the amount of new land converted. Because some lands are left fallow each year, comprehensively identifying existing cropland requires data from multiple years. We considered land that was cropped in any of the years 2006–2008 as existing cropland and only additional land cropped in 2009 as new cropland. This analysis suggests that approximately 200,000 ha was newly converted to cropland in Kansas in 2009, equivalent to 4.2 percent of the susceptible area per year.

Impacts from these three threat categories are cumulative, such that the total risk of development can be estimated by adding the risk of conversion from each category. However, our estimates suggest that the threat of conversion to cropland is two orders of magnitude higher than the threat of conversion to wind energy production or exurban development. Therefore, mitigation funds for protection should be targeted toward grasslands that are greater than 95 percent intact and that fall within the areas identified to be at risk of conversion to cropland.

#### Prairie-chickens

We recommend that impacts to optimal prairie-chicken habitat be offset through habitat restoration activities on existing intact grasslands. The greater prairie-chicken occurs in the northern and eastern portions of Kansas, and the lesser prairie-chicken occurs in the southern and western portions of the state, with some overlap in their ranges (Figure 2B). Preference should be given to in-kind offsets, so that impacts to greater prairie-chicken habitat should be offset with restoration of greater prairie-chicken habitat and impacts to lesser prairie-chicken habitat should be offset with restoration of lesser prairie-chicken habitat. Because lesser prairie-chickens are of higher conservation concern, we recommend that impacts within the area where the two species overlap be offset with restoration of lesser prairie-chicken habitat.

Altered fire return intervals, invasive species, and woody encroachment are considered major detriments to habitat quality for prairie-chickens [63]. We propose using mitigation funding to abate these threats on otherwise suitable prairie-chicken habitat. Targeting these restoration efforts to lands protected with conservation easements would ensure that the restoration efforts are not undone by future conversion of the restored lands. We estimated grassland restoration costs based on costs for tree removal and fire management, although invasive species control, range improvement, and other restoration activities may also be desirable to consider.

The ideal fire return interval for prairie chickens varies across tallgrass, mixed grass and short grass prairies. Ideal fire management for prairie-chickens in tallgrass prairie consists of prescribed burning approximately once every 3 years [64]. This fire regime is consistent with pre-settlement fire regimes and is favorable to many grassland-dependent birds [65]. In much of Kansas' tallgrass prairie, frequent burning adversely affects habitat structure resulting in reduced nesting success for greater prairie-chickens [63], [66]; thus, incentives for landowners to reduce fire frequency to once every 3 years are needed in these areas. In much of the rest of the state, fires are too infrequent, allowing woody plants to degrade habitat, thus incentives are needed to increase fire frequency. In mixed-grass prairie, a fire-return-interval of approximately once every 5 years is recommended; in shortgrass prairie, the ideal fire management consists of prescribed burning approximately once every 7 years (S.D. Fuhlendorf, personal communication). Recommendations presented here are of a general nature and specific management practices that would maximize habitat benefits would need to be tailored to specific properties.

#### Playas

Playa conservation typically requires acquisition (fee title or perpetual conservation easements) of playas and restoration of immediately adjacent grasslands. Most remaining playas in Kansas are in tilled agricultural fields, such that playa conservation requires restoring grasses and forbs around playas to restore hydrological function, reduce sedimentation, and limit fertilizer and pesticide runoff [67], [68]. Grassland restoration ratios are commonly established at three ha of grassland for each surface ha of the playa, based on the amount of grassland thought to be necessary to restore playa hydrological and ecological functions. Thus, for every hectare of high quality playa impacted, 1 ha of playa plus 3 ha of surrounding land (4 ha total) would be permanently protected, and the 3 ha surrounding the playa would be planted to native grasses and forbs. Because native grass and forb plantings constitute restoration of that playa, we consider all hectares of restored playa to be “additional.” Funds generated from impacted playa habitats should be used to protect playas that are of high or very high quality and are within playa complexes. Priority should be given to playas with documented whooping crane use.

#### Mitigation Costs for Existing Wind Development

We obtained data on the spatial location of 5,792 existing wind turbines in Kansas [54]. We determined whether each turbine is located in an area where we recommend avoiding wind development. For the turbines outside of avoidance areas, we identified clusters of turbines (i.e. turbines whose ecological footprints overlap) and calculated mitigation costs, if any, for each cluster.

## Results

Within Kansas there are approximately 14.5 million ha suitable for wind energy development (based on wind power class, distance to current and proposed transmission, and excluding urban and protected areas). If all of these areas were developed for wind energy, they could support approximately 668 GW of electrical capacity (WPC 3: 6,622,300 ha, 285 GW; WPC 4: 6,787,900 ha, 326 GW; WPC 5: 1,103,200 ha, 57 GW). After removing the wildlife avoidance areas that we identified, approximately 10.3 million ha remain as suitable for wind energy development. This “open” area is capable of yielding approximately 478 GW of electrical capacity (WPC 3: 4,478,500 ha, 193 GW; WPC 4: 5,012,700 ha, 241 GW; WPC 5: 857,300 ha, 45 GW). Even after removing both the wildlife avoidance areas and all areas where mitigation payments would be required, there are approximately 2.7 million ha suitable for wind energy development where no mitigation payments would be required (13 percent of the state). This area would be capable of supporting approximately 125 GW of electrical capacity (WPC 3: 1,366,000 ha, 59 GW; WPC 4: 1,193,700 ha, 57 GW; WPC 5: 175,500 ha, 9 GW). Note that the DOE goal for wind energy in Kansas is 7.16 GW, so even if all wind development was restricted to lands where no mitigation payment is needed, the wind capacity on these lands is 1,648 percent higher than (over 17 times higher than) the DOE goal.

### Identifying Areas Where Wind Energy Development Should Be Avoided

Avoiding Cheyenne Bottoms and Quivira National Wildlife Refuge and lands within a 16 km radius of each marsh removes 264,900 ha of economically viable wind from potential production. Avoiding all areas within 800 m of repeated whooping crane stopover sites removes 95,600 ha of economically viable wind from potential production. Avoiding very high quality playas within the whooping crane corridor by 800 m, removes 2,300 ha of economically viable wind from potential production. Avoiding very high quality playas outside the whooping crane migration corridor by 400 m removes another 5,000 ha of economically viable wind resource from potential production. Avoiding wind energy development within a 24 km radius of bat roosts and hibernacula in the Kansas Red/Gypsum Hills removes 216,300 ha of economically viable wind potential from production. Although this is a large area, only 50,963 ha of this area requires avoidance due to bats alone – the rest of it would need to be avoided due to intact grasslands or whooping crane stopover sites, regardless of bats. Avoiding grasslands that are 95 percent intact, plus a 1.6 km buffer around intact grasslands that overlap with optimal prairie chicken habitat, removes 3,821,000 ha of economically viable wind resource from potential production.

### Quantifying Impacts that need to be Offset

**Figure 4:**
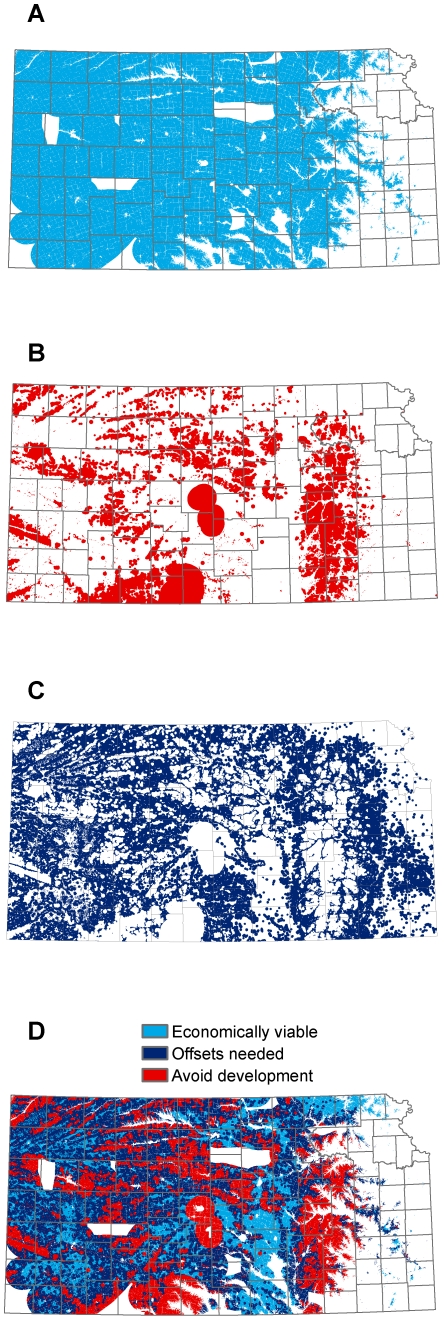
Avoidance and mitigation areas in Kansas. A) areas of potential wind development, B) avoidance areas, C) areas requiring mitigation, and D) all three layers, with avoidance and mitigation areas superimposed over areas of potential wind development; remaining light blue areas indicate areas suitable for wind development where mitigation would not be required.

We identified 7.6 million ha outside of avoidance areas where development could proceed but where sensitive resources requiring compensatory mitigation exist (Figure 4). These sensitive resources are: 1) areas with 50–95 percent intact grasslands, 2) optimal prairie-chicken habitat, and 3) playas of less than very high quality. There are 7,203,000 ha of economically viable wind resource on intact grasslands that would require mitigation payments. This includes 1,660,900 ha of economically viable wind resources that would also require compensatory mitigation for prairie-chickens. For impacts to playas outside of the whooping crane migration corridor, there are 540,600 ha of economically viable wind where development would require compensatory mitigation. For impacts to playas inside of the whooping crane migration corridor, there are 278,000 ha of economically viable wind resource where development would require compensatory mitigation.

### Quantifying Compensatory Mitigation Costs

#### Intact Grasslands

Mitigation costs for impacts to shortgrass, mixed-grass and tallgrass habitats would be $825, $862, and $1,432 per ha of impact, respectively. We estimate that a successful offset program would protect lands that have, on average, an annual risk of conversion of 4.2 percent. Thus, protecting 100 ha of grassland under this program would result in 84 ha of avoided conversion after 20 years. Because development impacts begin immediately, whereas the benefits of avoided conversion accrue more slowly over time, we apply a temporal discounting factor of 3 percent per year to the benefits of avoided conversion. Based on this discounting and the annual risk of conversion, we calculate the benefit of protecting 100 ha to have a net present benefit equivalent to 60 ha. This means that no net habitat loss from wind energy projects requires protection of 1.67 ha for every hectare impacted. We calculate the price of land protection via perpetual easement at $494, $516, and $858 per ha for shortgrass, mixed-grass, and tallgrass habitats, respectively [69]. Because 1.67 ha need to be protected for every ha that is impacted, we multiply these easement prices by 1.67 to obtain the costs for each ha of impact. Conservation easements provide legal protection for land that is already good habitat, such that no discounting for the probability of success is required.

#### Prairie-chickens

For prairie chicken mitigation, we calculate the costs to: 1) restore and maintain natural fire return intervals, and 2) remove and prevent woody and other plant encroachments. Restoring fire return intervals to benefit prairie-chickens could be incentivized by paying to conduct prescribed burns. We estimate that prescribed burning costs $13 per ha ($5.25 per acre, based on WHIP NRCS rates for Kansas [70]). Financial endowments sufficient to generate $13 per hectare every three, five, or seven years would require an initial investment of $82, 47, and $32 per hectare, respectively, assuming an average annual interest rate of 5 percent. Note that to receive payments landowners would be required to burn at the prescribed return interval, which may be more or less frequent than current practice. An additional one-time payment of $214 per ha for brush management (average of medium and low mechanical brush management treatment costs, based on WHIP NRCS rates for Kansas) [70] should be added to the prescribed fire endowment to complete the mitigation offset.

Summing the costs of brush removal and the prescribed fire endowment, the total cost per ha is $296 for tallgrass prairie, $261 for mixed grass, and $246 for short grass. Habitat benefits from brush removal and prescribed fire occur consistently and rapidly after implementation, such that no temporal or probability of success discounting is required for these offset activities.

#### Playas

Offsets for impacts to playas will require restoring native grasses and forbs around existing playas and purchasing protection rights (via fee title or perpetual conservation easements) on both the playas and surrounding restored habitat. For each hectare of playa that is impacted by the wind energy development, 4 ha would be protected and 3 ha of these protected lands would be restored. Planting native grasses and forbs costs about $208 per ha, so that the 3 ha of required restoration for each ha of impact can be accomplished for $624. Average fee title prices in areas with high and very high quality playa lakes are $3,062 per ha ($1,239 per acre). Therefore, each hectare of high quality playa would require $12,872 of mitigation payments, including the restoration and protection of the grassland buffer. Based on their lower ecological function (i.e. supporting fewer migratory birds on average across years), we assess that medium quality lakes require only 66 percent of the per-hectare mitigation costs for high quality lakes. For the same reasons, low quality playa lakes only require mitigation if they are a part of a multi-lake complex, and then only at 33 percent of the per-hectare mitigation cost. The benefits of replacing cropland with grassland are consistently realized quickly, such that no temporal or probability of success discounting is required.

#### Mitigation Costs for Existing Wind Development

**Figure 5:**
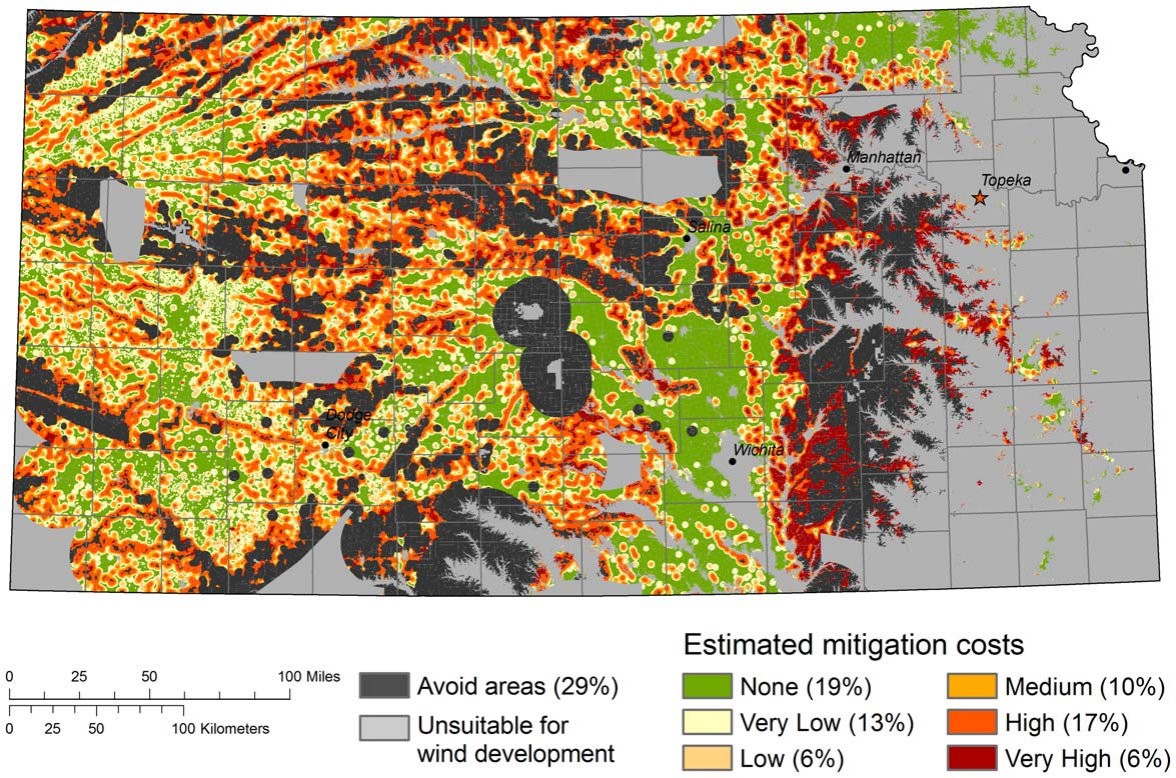
Relative mitigation cost surface for wind development in Kansas. Relative mitigation costs were calculated only for areas suitable for wind outside of recommended avoidance areas. The percent of the total area suitable for wind is shown in parentheses for each category.

For existing wind turbines, 15 percent are located in areas that we recommend be avoided and 19 percent are located in areas that would require no mitigation payments. Omitting turbines that are in avoidance areas, we found that the remaining turbines occurred in 128 clusters, where clusters contained turbines with overlapping ecological footprints. Of these, 21 clusters would not require any mitigation payments. For the remaining 107 clusters that did require offsets, the average per turbine cost of mitigation was $32 thousand dollars and the median cost was $23 thousand dollars. The cost of wind turbine development is roughly $4 million dollars per turbine, so the median cost of mitigation is roughly equal to 0.57 percent of development costs. Because the cost of mitigation varies greatly depending on project and turbine siting (Figure 5), developers can reduce mitigation costs by siting future development in areas with low mitigation costs.

## Discussion

Our results are intended to facilitate ecologically appropriate siting of wind energy development, while ensuring that key ecological targets are conserved. The increase in wind energy production forecasted by DOE for Kansas may be compatible with wildlife needs, if commercial wind energy facilities are properly sited. For example, many of the tilled agricultural areas within the state represent low-quality habitats incapable of supporting populations of imperiled species or natural plant or animal communities. New wind energy development would likely have substantially less potential to impact wildlife if sited in these areas [71].

Our analysis indicates that a network of land-based turbines has the potential to generate 478 GW of capacity on 10.3 million ha in Kansas, even after removing areas incompatible with conservation, areas with low wind speeds, and areas far away from transmission lines. This represents 6,674 percent of the DOE projection of 7.16 GW for Kansas needed to generate 20 percent electricity from wind by 2030. The fact that 85 percent of existing wind turbines are sited outside of areas incompatible with conservation further supports our argument that it is possible to develop wind energy without compromising conservation goals. Even after removal of all lands that would require compensatory mitigation for impact, there is still an ample land base of 2.7 million ha that can more than meet the DOE projections. Even if this land base were restricted to the highest WPC (i.e. WPC 5 in Kansas), this leaves 175,500 ha capable of siting 9 GW of wind, more than enough to meet the DOE goal.

Our approach describes ecologically important areas in Kansas where wind energy development impacts could not be offset. Our criteria for avoidance by wind energy development are based on the best available science regarding known high priority conservation targets in Kansas. Our analysis follows best practices for conservation planning [18], by considering multiple conservation targets designed to preserve both whole landscapes and particularly sensitive areas, including: key habitats (intact grasslands and playas); umbrella species (greater and lesser prairie-chickens); particularly imperiled species (whooping crane); and areas of wildlife congregation for taxa that may be particularly vulnerable to wind energy impacts (bat roosts and playas). We recognize that other approaches are possible and that better data would allow refinement of these avoidance areas, but we suggest that any comprehensive conservation planning approach would yield qualitatively similar conclusions about the location of the most sensitive habitats in Kansas.

Our approach to mitigation estimates the costs that would be required to offset the impacts of a particular project to achieve no net loss of habitats. In order to calculate these costs, we identified conservation strategies for application of mitigation dollars. Our strategies seek to provide high returns on investment, such as conservation easements and restoration practices that can be implemented by landowners with modest conservation payments. By pooling funds to achieve economies of scale and by facilitating strategic application of these funds, conservation outcomes are maximized, while mitigation costs for developers are reduced. The conservation practices and the spatial analyses used to select areas where practices would best be applied are intended to aid the strategic use of mitigation funds; they are not intended to constrain innovation or other opportunistic use of mitigation funds. Thus, our analyses describe minimum conservation benefits that can be achieved with the specified mitigation funds; strategic and innovative application of these funds could result in conservation benefits beyond those identified here.

Costs for mitigation actions described here could often be incorporated into the business costs of developing wind energy, given that the overall investment for a commercial wind energy facility is commonly hundreds of millions of dollars. We find that the median cost of mitigation is roughly half a percent of per turbine development costs. More importantly, wind energy developers can use the results of this analysis to proactively reduce the need for mitigation by siting projects in areas that would not warrant mitigation. This could substantially reduce the cost of mitigation across projects. For example, although we recognize that the costs per ha for playa impacts are noticeably higher than for impacts to intact grasslands and prairie-chickens, they are also easier to avoid because 1) the ecological footprints of wind turbines are smaller for playa impacts, 2) playas comprise a small percentage of the land area in Kansas (only 0.15 percent), and 3) playas are relatively small (median playa size is 0.67 ha), often allowing impacts to playas to be avoided through micro-siting of individual turbines.

In addition to avoiding and offsetting impacts, operational mitigation may be employed to reduce direct mortality impacts to some susceptible bats and birds. Ongoing research is evaluating the possibility of operational mitigation strategies to minimize mortality by feathering turbine blades (which stops the blades from spinning) at critical periods [72], [73]. Bat fatalities occur during predictable times: at night, mostly during fall migration, and when wind speeds are below 6 meters per second [42]. Bat fatalities often coincide with particular weather conditions, e.g. when bats migrate with storm fronts. This suggests that radar and other remote sensing technology systems could be developed to detect bat or bird migrations in real time, allowing blades to be feathered as needed to minimize fatalities.

**Figure 6:**
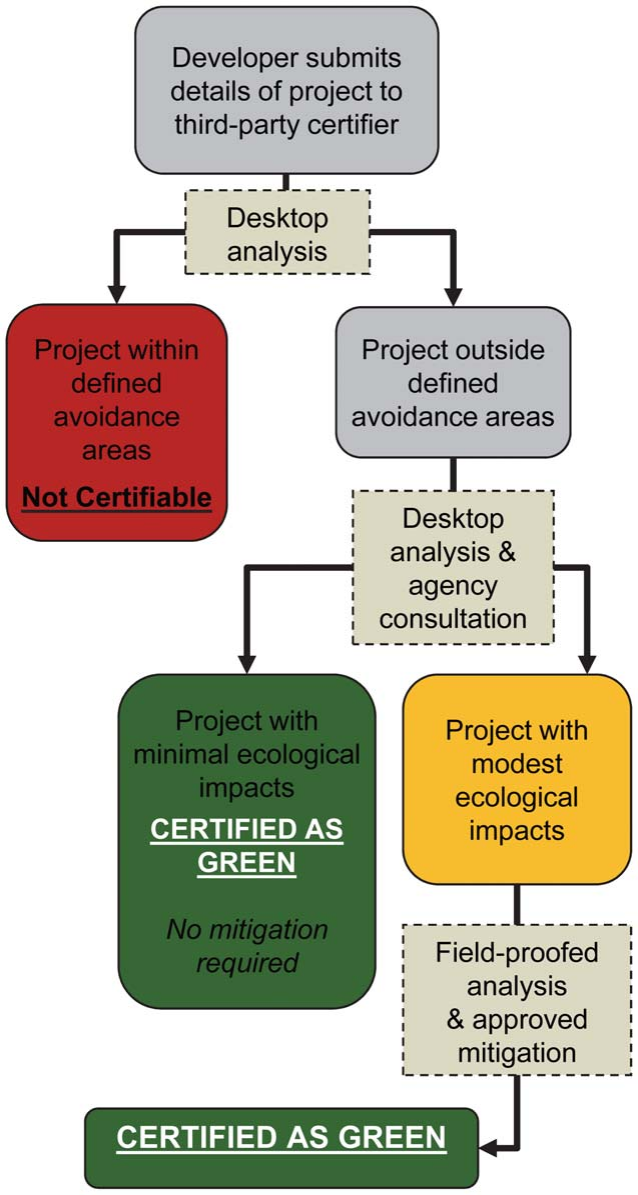
Schematic showing proposed steps of a Green Certification process for wind energy development.

Our research illustrates that it is presently possible to implement a landscape-scale system that guides wind energy development to avoid, minimize, and offset ecological impacts. The approach outlined here, updated with new information as it becomes available, could be used to award “Green Certification” to projects that follow this protocol. The steps that would be necessary to achieve certification are illustrated in Figure 6. Certification against the guidelines presented in this paper may help to expand and sustain the wind industry by facilitating the completion of individual projects sited to avoid sensitive areas and protecting the wind industry's reputation as an ecologically friendly source of electricity. Endorsement of a Green Certification process by electric utilities and financial backers would provide incentives for wind developers to seek certification for new facilities.
